# National trends in incidence and geographic distribution of melanoma and keratinocyte carcinoma in the Russian Federation

**DOI:** 10.3389/fmed.2023.1188038

**Published:** 2023-07-20

**Authors:** Anastasiya Muntyanu, Feras M. Ghazawi, Elena Pastukhova, Andrei Zubarev, Ivan V. Litvinov

**Affiliations:** ^1^Division of Dermatology, McGill University, Montreal, QC, Canada; ^2^Division of Dermatology, University of Ottawa, Ottawa, ON, Canada; ^3^Faculty of Medicine, University of Ottawa, Ottawa, ON, Canada

**Keywords:** melanoma, Non-Melanoma Skin Cancer, keratinocyte carcinoma, incidence, risk factors, Russian Federation

## Abstract

Keratinocyte Carcinomas (KC), including basal cell and cutaneous squamous cell carcinomas, are the most common skin cancers in Fitzpatrick phototype I-III individuals, while melanoma is one of the deadliest skin cancer types. The incidence of both melanoma and KC is increasing in Russia. KCs’ incidence increases from north-to-south across the Russian Federation. In contrast, while melanoma’s incidence increases from north-to-south in the eastern part of the country, in the west of Russia a reverse latitude gradient trend is noted, where northern more affluent regions of Russia display higher rates of melanoma than the southern jurisdictions. Furthermore, our detailed analysis of incidence by jurisdiction highlights that affluent northern capital cities have higher rates of melanoma than the surrounding regions. The observed melanoma incidence trends in the western portion of Russia are similar to the findings in the western Europe and opposite of the findings in Canada.

## Introduction

The incidence of both melanoma and keratinocyte carcinomas (KCs), including basal cell carcinoma (BCC) and cutaneous squamous cell carcinoma (cSCC), has been increasing over the last several decades (1–3). KCs, also known as Non-Melanoma Skin Cancers (NMSC), are the most common cancers affecting fair-skinned individuals and include BCCs and cSCCs (4). Some of the risk factors for these cutaneous malignancies include a fair skin phototype, ultraviolet radiation (UVR) exposure, history of sunburn, genetic factors/mutations (e.g., mutations in the MC1R gene) and photoprotective behaviors (5–9).

The Russian Federation is a large, diverse country with various environmental, geographic, cultural zones making it a suitable location to study risk factors and trends for cutaneous malignancies. Exploring the geographic distribution of melanoma vs. KC helps highlight critical differing risk factors for each malignancy as well as detail high-risk skin cancer areas that could benefit from increased screening and counseling on photoprotective behaviors.

## Methods

We recently retrospectively analyzed the data from the Moscow Oncology Research Institute, a national, publicly available, cancer registry. Data was extracted based on corresponding ICD-10 codes for years 2001–2017 for melanoma (C43) and 2007–2017 for KC (C44) as well as other cancers, by year and jurisdiction (80 jurisdictions in Russia), as described in detail in our previous studies (5, 10, 11). For diagnosis of BCCs and cSCCs in Russia, a biopsy is required, hence the results are tracked in the registry. For annual age-standardized incidence rate (ASIR), the WHO World Population Standard 2000–2025 was used. For analysis by jurisdiction, the crude incidence rates were reported. The data on regional population was extracted from the Russian Federation Census for the years 2002 and 2010. Linear regression analysis was completed to assess trends in incidence over time. Risk factors assessed in descriptive analyses included UVR, skin phototype, cultural sun protection/clothing practices, and climate zones. Additionally, Pearson correlation was completed to determine relationship between melanoma/KC incidence rates and Gross Domestic Product (GDP) *per capita*, or the total number of physicians and dermatologists per region.

## Results

**Figure 1:**
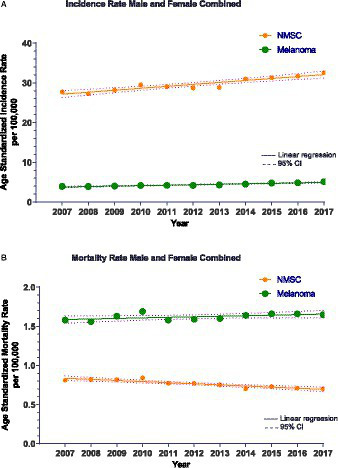
**(A)** Non-Melanoma Skin Cancer (NMSC)/Keratinocyte carcinoma (KC) incidence for all cases during 2007–2017 with the line of best fit and linear regression analysis. *R*^2^ = 0.87 and *p* < 0.0001. The slope of the line is 0.49 cases/100,000 person-years. Melanoma incidence for all cases during 2007–2017 with the line of best fit and linear regression analysis. *R*^2^ = 0.91 and *p* < 0.0001. The slope of the line is 0.12 cases/100,000 person-years. Only years 2007–2017 depicted here for comparison with CM. CI, Confidence interval. **(B)** Non-Melanoma Skin Cancer (NMSC)/Keratinocyte carcinoma (KC) mortality during 2007–2017 with the line of best fit and linear regression analysis. *R*^2^ = 0.83 and *p* < 0.0001. The slope of the line is −0.014 cases/100,000 person-years. Melanoma mortality during 2007–2017 with the line of best fit and linear regression analysis. *R*^2^ = 0.30 and *p* = 0.037. The slope of the line is 0.007 cases/100,000 person-years. CI, Confidence interval.

Our combined results demonstrated that a total of 141,597 patients (62% females) were diagnosed with melanoma during 2001–2017 with age-standardized incidence (ASIR) and mortality (ASMR) rates of 4.27/100,000 and 1.62/100,000, respectively. For KC, 733,723 patients (63% females) were diagnosed during 2007–2017 and corresponding incidence and mortality rates were 29.64/100,000 and 0.70/100,000, respectively. For both melanoma and KC, increasing annual incidence rate of 0.096 cases/100,000 (*p* < 0.0001) and 0.49 cases/100,000 individuals per year (*p* < 0.0001), respectively, was observed (Figure 1A). While for melanoma there was an increasing mortality trend, for KC a decreasing trend was noted (Figure 1B).

Melanoma incidence was not significantly correlated with GDP (*p* = 0.94), and the total number of physicians (*p* = 0.087) or dermatologists (*p* = 0.12) per jurisdiction. KC incidence was negatively correlated with GDP and reached statistical significance (*p* = 0.004), while it was not significantly associated with the number of physicians (*p* = 0.910) or dermatologists (*p* = 0.887) per jurisdiction.

Analysis by proportion of skin phototypes revealed a higher proportion of Fitzpatrick I/II individuals in the top 10 high-incidence regions for melanoma (95.13%) and KC (88.6%) compared to the lowest 10 incidence regions (36.3% vs. 50.1%), respectively.

**Figure 2:**
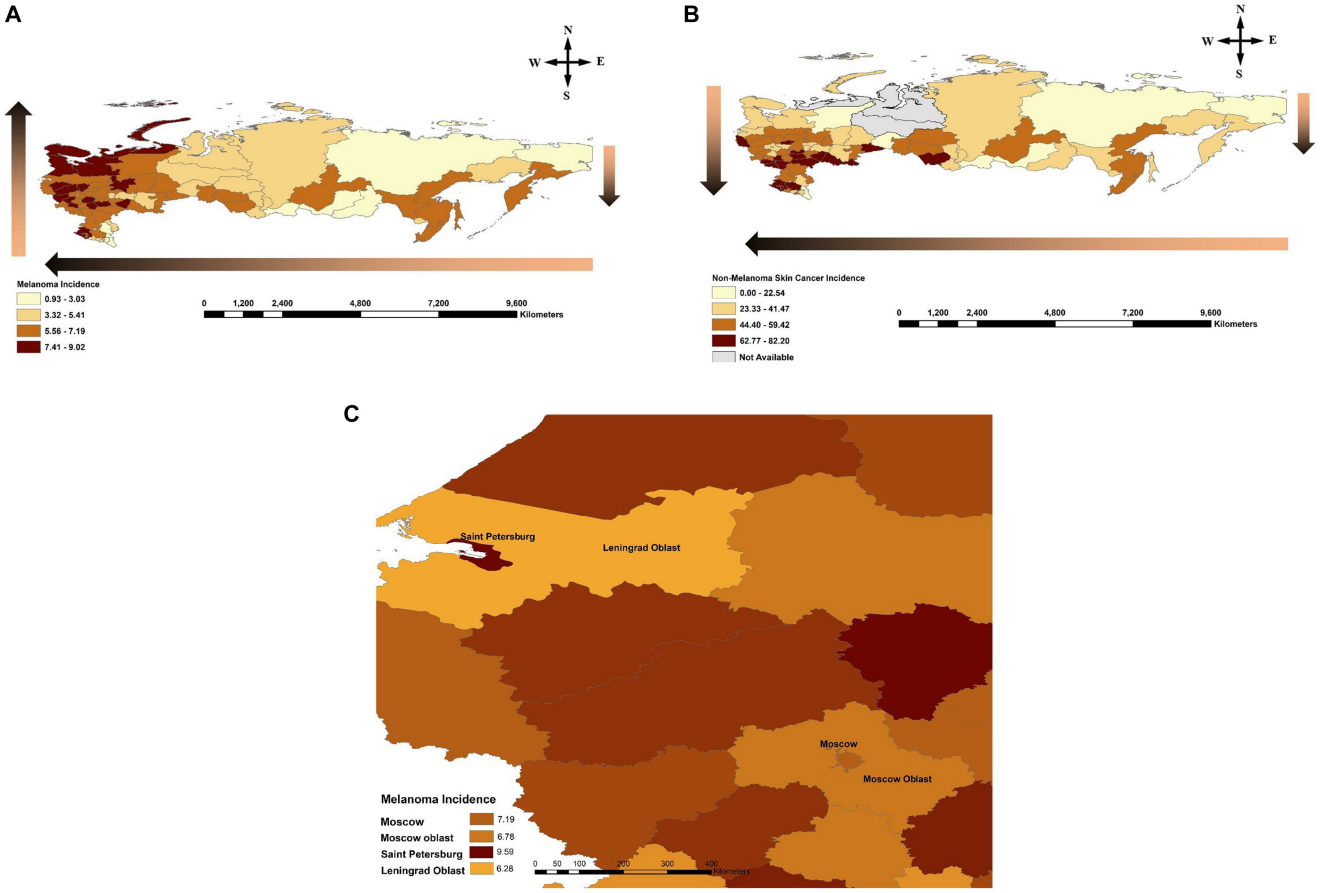
**(A)** Melanoma incidence across the Russian Federation by jurisdiction during 2007–2017. Gradient arrows show a reverse south-to-north trend in the western part of the country compared to the north-to-south trend observed in the east, and increasing incidence from east-to-west. **(B)** Non-Melanoma Skin Cancer (NMSC)/Keratinocyte Carcinoma (KC) incidence across the Russian Federation by jurisdiction during 2007–2017. Gradient arrow shows a north-to-south and east-to-west trends of increasing incidence across the country. **(C)** Comparison of incidence rate between capital cities and corresponding surrounding jurisdictions for Saint Petersburg/Leningrad Oblast as well as Moscow City/Moscow Oblast. Incidence is reported per 100,000 individuals per year by federal jurisdiction.

For melanoma and KC, a decreasing incidence from west to east was observed. We observed a north-to-south gradient in incidence across Russia for KC. For melanoma, a north-to-south gradient in incidence was present in the eastern part of the country. In contrast, in the western part of Russia, a reverse south-to-north gradient in melanoma incidence was noted (Figures 2A,B). Additional analysis comparing capital cities to the surrounding oblast/region (e.g., Saint Petersburg vs. Leningrad oblast, Moscow vs. Moscow oblast) revealed higher incidence rates in these cities compared to the adjacent jurisdictions (Figure 2C).

## Discussion

Annual increasing incidence trends over time have been observed in Russia for both melanoma and KCs, similar to global trends (10). Notably, in our study the incidence of melanoma and KC for women was higher than for men, contrary to the trends reported in other parts of the world. This could be attributed to cultural perspectives in Russia where many Caucasian females strive to attain a darker skin tone and thus, inadvertently, increase the cumulative UV exposure placing them at risk of skin cancer. Additionally, it is possible that females are more likely to consult their physician regarding a skin lesion leading to a higher rate of detection. However, these hypotheses require further investigation. In our study, the decreasing mortality for KC could be attributed to improved and earlier detection and treatment.

In the eastern part of Russia both cancers have lower incidences in the northern regions and higher incidences in the southern jurisdictions (Figures 2A,B). For KC, this trend remains true in the western part of Russia as well. However, for melanoma, in the western part of Russia incidence demonstrates a reverse south-to-north (reverse latitude) gradient. This could be due to increased affluence in the north and more opportunities for vacation time in warmer climates as well as tanning bed use, leading to intermittent high UV exposure which is atypical for Caucasian individuals’ skin living in this climate. Strikingly, when Saint Petersburg was compared to Leningrad oblast (Saint Petersburg is one of the capital cities of Russia embedded in a Leningrad oblast) and Moscow to Moscow oblast, higher incidence rates of melanoma were observed in these capital cities compared to the surrounding regions despite similar geography (Figure 2C). Hence, increased affluence in the capital cities of Russia may be contributing to increased UV exposure and higher incidence of melanoma.

Another important risk factor is a skin phototype impacting melanoma incidence differently than KC. We noted that in the Republic of Dagestan, Ingushetia and Chechen Republic (in the south of the country) there was a lower incidence of melanoma. These regions are largely inhabited by individuals with Fitzpatrick phototypes III/IV skin. Additionally, there is a higher proportion of Muslim faith practicing individuals who typically have more conservative dress practices covering the skin and, hence, minimizing the risk of UVR exposure resulting in even fewer cases of melanoma. In contrast, incidence of KCs remains high in the southern regions of the country, perhaps, due to chronic exposure to increasing UVR levels at lower latitudes being a dominant risk factor for NMSC/KC rather than a strong intermittent exposure in the north. Of note, Saint Petersburg city (located in the north) which had the highest rate in the country for melanoma was ranked much lower (61/80) in incidence for KC.

Our findings are in keeping with other studies that demonstrated generally increasing skin cancer rates in Caucasian populations toward the equator due to increase in direct UVR (12) with a notable reversal of the trend in western Europe. In this region, individuals with lighter skin phototype and increased affluence residing in the north have higher melanoma risk than population with darker skin tones (e.g., Italy, Spain, and Portugal) residing in the south (13). Interestingly, this is also opposite to the trend observed in Canada (also a large, multicultural country, spanning similar latitudes), where increasing rates of melanoma were observed toward the south (i.e., the expected latitude gradient) (3, 14–16). The separating factor here is that there is no increased affluence in the north of Canada and, hence, lower intensity UVR combined with darker skin phototype of Indigenous populations residing in the north remain the predominant protective factors (2, 16).

Finally, for both cutaneous malignancies, an east to west gradient of increasing incidence was observed (Figures 2A,B). This could be explained based on the climate zones where more circumpolar regions are in the east and more tropical regions are in the west, favoring more outdoor time, which is similar to our findings in Canada (2). Additionally, skin phototype generally changes from darker to lighter subtypes moving from the east of Russia westward, increasing the effect UVR has on the skin. The importance of skin phototypes is supported by the observation of increased proportion of Fitzpatrick I/II individuals in the top 10 high-incidence incidence regions compared to the lowest 10 incidence skin cancer areas in the country.

Given that there was a negative association between KC incidence and GDP, this suggests that perhaps increased counseling and access to photoprotective measures contribute to lower KC rates in areas with higher GDP. On the other hand, no such association was noted with melanoma incidence.

This was a large populational study in the Russian Federation assessing malignancy trends. Several limitations exist for this approach, as previously discussed (5, 10, 11). Notably, life expectancy data for each region was not available which may impact rates detected if there is a larger proportion of older individuals living in a given jurisdiction.

In conclusion, based on the findings of this study, skin phototype, UVR levels and recreational/cultural practices were the three most important risk factors. Additionally, intermittent exposure to high UVR is a critically important risk factor for melanoma while chronic UVR exposure for KC. Hence, advocacy for sun protection, improved screening programs and minimization of tanning bed use are critical to reduce the rates of these cancers in the future.

## Data availability statement

The original contributions presented in the study are included in the article/supplementary material, further inquiries can be directed to the corresponding authors.

## Author contributions

AM: conceptualization, methodology, data curation, formal analysis, investigation, and visualization. FG: data curation, formal analysis, funding acquisition, visualization, and investigation. EP: data curation, formal analysis, and investigation. AZ: conceptualization, methodology, funding acquisition, data curation, formal analysis, investigation, visualization, and writing—review and editing. IL: resources, supervision, investigation, methodology, project administration, and writing—review and editing. All authors contributed to the article and approved the submitted version.

## Conflict of interest

The authors declare that the research was conducted in the absence of any commercial or financial relationships that could be construed as a potential conflict of interest.

## Publisher’s note

All claims expressed in this article are solely those of the authors and do not necessarily represent those of their affiliated organizations, or those of the publisher, the editors and the reviewers. Any product that may be evaluated in this article, or claim that may be made by its manufacturer, is not guaranteed or endorsed by the publisher.
